# Parishin alleviates vascular ageing in mice by upregulation of Klotho

**DOI:** 10.1111/jcmm.17740

**Published:** 2023-04-09

**Authors:** Xinxiu Zhao, Shixian Zhou, Yang Liu, Caixia Gong, Lan Xiang, Shumin Li, Peixia Wang, Yuejun Wang, Linlin Sun, Qin Zhang, Yunmei Yang

**Affiliations:** ^1^ Department of Geriatrics, The First Affiliated Hospital, School of Medicine Zhejiang University Hangzhou China; ^2^ Key Laboratory of Diagnosis and Treatment of Aging and Physic‐chemical Injury Diseases of Zhejiang Province, The First Affiliated Hospital, School of Medicine Zhejiang University Hangzhou Zhejiang China; ^3^ College of Pharmaceutical Sciences Zhejiang University 866 Yu Hang Tang Road Hangzhou China; ^4^ Zhejiang Aged Care Hospital Hangzhou Normal University Hangzhou Zhejiang China

**Keywords:** ageing, FoxO1, Klotho, network pharmacology, parishin, senescence, vascular endotheliocyte

## Abstract

Senescence of vascular endothelial cells is the major risk of vascular dysfunction and disease among elderly people. Parishin, which is a phenolic glucoside derived from *Gastrodia elata*, significantly prolonged yeast lifespan. However, the action of parishin in vascular ageing remains poorly understood. Here, we treated human coronary artery endothelial cells (HCAEC) and naturally aged mice by parishin. Parishin alleviated HCAEC senescence and general age‐related features in vascular tissue in naturally aged mice. Network pharmacology approach was applied to determine the compound‐target networks of parishin. Our analysis indicated that parishin had a strong binding affinity for Klotho. Expression of Klotho, a protein of age‐related declines, was upregulated by parishin in serum and vascular tissue in naturally aged mice. Furthermore, FoxO1, on Klotho/FoxO1 signalling pathway, was increased in the parishin‐intervened group, accompanied by the downregulated phosphorylated FoxO1. Taken together, parishin can increase Klotho expression to alleviate vascular endothelial cell senescence and vascular ageing.

## 1 INTRODUCTION

Senescent cells evade immune clearance, accumulate in human tissues during ageing and contribute to age‐related pathologies.^1^, ^2^ Endothelial cell senescence leads to a vascular dysfunction and induces vascular diseases in aged population.^3^ Vascular system, one of the most critically systems, will drive physiological dysfunctions of other organs to elevate the incidence of morbidity and mortality.^4^ Therefore, finding effective interventions for the prevention of vascular ageing is a very important field for geriatric medicine.

Tianma (*Gastrodia elata* Blume) is an herbal medicine that has been widely used in East Asia for hundreds of years. It has unique medicinal effects for the treatment of headache, dizziness and stroke, and also has anti‐inflammatory and antioxidant effects.^5^, ^6^ Parishin is a phenolic glucoside isolated from *G. elata*. Lin et al. found that parishin significantly prolonged yeast lifespan, increased Sir2 gene expression and inhibited the UTH1/TOR signalling pathway through antioxidant stress.^7^ This study suggested that parishin had anti‐ageing effects. Thus, parishin may be a valuable lead compound for the pharmacological treatment of age‐related diseases. However, the effect of parishin on endothelial cell senescence and vascular ageing has not been explored.

Network pharmacology approach, providing the compound‐target networks for traditional Chinese medicine (TCM), found that parishin had a strong binding affinity for Klotho. Klotho is an anti‐ageing protein that can attenuate ageing‐related diseases to extend healthspan.^8^, ^9^ α‐Klotho declines with ageing in mice and humans.^10^ Overexpression of α‐Klotho increased lifespan and delayed ageing‐related vascular dysfunction.^10^, ^11^ FoxO1 is a member of the forkhead transcription factor O subfamily, which controls various biological processes (BPs), including oxidative stress, metabolic regulation, cell proliferation and apoptosis.^12^ Phosphorylated FoxO1 will translocate into cytoplasm and be degraded in proteasomes.^13^ Klotho promoted translocation of FOXO1 from the cytoplasm to the nucleus by reducing FoxO1 phosphorylation.^14^

Here, we investigate whether parishin could target on Klotho to ameliorate vascular endothelial cell senescence and vascular ageing in naturally aged mice. We found that parishin rescued ageing phenotypes in senescent human coronary artery endothelial cells (HCAEC) and aged vascular tissues by increasing the level of Klotho. Furthermore, Klotho maintained FOXO1 by the downregulation of phosphorylated FoxO1. Our study points to a promising therapeutic potential of parishin in reducing vascular diseases in the elderly.

## 2 MATERIALS AND METHODS

### 2.1 Cell culture

Primary HCAECs (SCIENCELL) were obtained and cultured using an endothelial cell medium (SCIENCELL) containing with 1% endothelial cell growth supplement and 5% foetal bovine serum. The culture medium was changed every 24 h. When HCAECs were 70%–80% confluent, the cells were trypsinized, resuspended in the culture medium and seeded into 96‐, 24‐, or 6‐well microplates for each assay.

Endothelial cell replicative senescence was studied by subjecting endothelial cells to subsequent passages until passage 16 HCAECs were plated at a seeding density of 2500 cells/cm^2^ in T25 flasks and the medium was changed every 48 h. Cell cultures reached confluence after 6–7 days, as assessed by light microscopic examination, and they were passaged at weekly intervals. After trypsinization and before replating, harvested cells were counted using the Trypan blue viability stain. Cell senescence was evaluated by SA‐β‐gal staining, and the protein levels of γH2AX PARP‐1, P16^Ink4a^ and IL‐6 were assessed by western blotting at the indicated population doubling level.

### 2.2 Drug administration

Passage 13 (P13) was selected as the starting point of parishin intervention according to the pre‐experiment results. Parishin powder was dissolved in DMSO and diluted into the final concentration of 3, 10 and 30 μM by medium for cell intervention. A control group was set to add the corresponding dose of DMSO. The cells were randomly divided into blank control group, parishin low‐concentration intervention group (3 μmol/L parishin), and parishin medium‐concentration intervention group (10 μmol/L parishin), parishin high‐concentration intervention group (30 μmol/L resveratrol). Cells were incubated for 3–4 weeks using the corresponding preformulated media as described above.

### 2.3 Animals

Parishin was provided by Qi Jianhua's research team at Zhejiang University (patent no. of Zhejiang University CN201610061288.2). Nineteen‐month‐old specific pathogen‐free healthy male C57B/L6 mice (*n* = 40) were purchased from Zhejiang Laboratory Animal Center. All mice were fed with normal diet and housed under a 12‐h light and 12‐h dark cycle (7 AM and 7 PM, 25°C and 70%–80% humidity) at the Laboratory Animal Center of the Medical Department of Zhejiang University. After a 2‐week acclimation period, the mice were randomly divided into four groups (*N* = 10 each group): aged control group (AC), aged low‐dose parishin treatment mice (LPar, parishin‐10 mg/kg/day), aged middle‐dose parishin treatment mice (MPar, parishin‐20 mg·kg1·d1) and aged high‐dose parishin treatment mice (HPar, parishin‐30 mg·kg1·d1).The parishin was dissolved in 0.9% saline (containing 0.5% DMSO) and administered orally by gavage to the parishin group daily, while the AC group were administered the same volume of 0.9% normal saline each day. The body weight of the mice was recorded every week. At Week 8, the mice were euthanized to collect serum, blood vessel and other tissues for further analysis. All experiments were conducted following ‘The Instructive Notions with Respect to Caring for Laboratory Animals’ issued by the Ministry of Science and Technology of the People's Republic of China. The study protocols were approved by the Committee on the Ethics of Animal Experiments of Zhejiang University (approval no. 2020; experimental Kuaishen no. 1446).

### 2.4 Human serum collection

After obtaining informed consent, participants were subjected to fasting conditions for at least 12 h. Blood samples were drawn from the cubital vein of subjects into 10 mL sterile drying tubes. Then, samples were allowed to clot for 2 h at room temperature before centrifugation for 15 min at 1000 × g. Freshly obtained serum samples were immediately processed or stored in aliquots for later use at −80°C.

### 2.5 Senescence‐associated beta‐galactosidase activity assay

Senescence‐associated beta‐galactosidase (SA‐β‐gal) activity was measured according to the manufacturer's protocol. Briefly, HUVECs were washed three times in phosphate‐buffered saline (PBS), fixed for 15 min at room temperature by the fixative solution, and incubated overnight at 37°C with fresh SA‐β‐gal stain solution at pH 6.0 (Thermo Fisher Scientific K145501). The percentage of SA‐β‐gal was calculated by counting the positively stained cells within a sample of 200 cells (100× magnification).

### 2.6 DNA damage detected with γ‐H2AX immunofluorescence

DNA double‐strand breaks in chromatin were detected using γ‐H2AX immunofluorescence. Briefly, cells were blocked with a specific blocking buffer for 1 h at room temperature in a humidified chamber, and then the cells were incubated with the primary antibody anti‐γ‐H2AX (ab 11174，1:200 dilution) overnight at 4°C. After washing the cells with PBS containing 0.15% Triton X‐100, the cells were incubated for 1 h at room temperature with fluorescently tagged secondary antibodies (1:1000 dilution), followed by a 5‐min incubation with DAPI to identify nuclei. Subsequently, the cells were covered by an anti‐fluorescent quencher and observed under a laser confocal scanning microscope (LSM 710) at an excitation wavelength of 594 nm. The secondary antibody was goat anti‐rabbit IgG H&L (DyLight® 594) (ab96885).

### 2.7 Cellular reactive oxygen species (ROS) detection

Cellular ROS in cells was detected using a ROS detection kit (S0033, Beyotime) as per the instructions. Briefly, 2′,7‐dichlo‐rodihydrofluorescin diacetate (DCFH‐DA) was diluted with MEM to a final concentration of 10 μmol/L. The cells were collected and suspended in diluted DCFH‐DA at a concentration of 1 × 10^6^ cells/mL and incubated at 37°C in a cell incubator for 20 min. The cells were washed thrice with MEM to remove DCFH‐DA that did not enter the cells. The ROS was detected under a laser confocal scanning microscope (LSM 710) at 488 nm.

### 2.8 Mitochondrial membrane potential staining assay

The cells were isolated and cultured to a certain density in a culture dish. After removing the culture medium, the cells were incubated with Mito‐Tracker Red CMX Ros working solution (C1049‐50 μg, Beyotime) at 37°C for 30 min. The Mito‐Tracker Red CMXRos working solution was removed, and pre‐incubated DMEM was added. Finally, the mitochondria were observed under a laser confocal scanning microscope (LSM 710) at 594 nm.

### 2.9 Western blot analysis

Protein concentration was determined using a BCA protein assay kit (Beijing Kangwei Century Biotechnology Co., Ltd.). Equal amounts of proteins (30μg) were placed in 4%–20% SDS‐PAGE gel (GenScript Biotechnology) and transferred to PVDF membrane (Bio‐Rad) by semi‐wet transfection for 15 min. The membranes were blocked with the Quickblock fast blocking solution (Beyotime Biotechnology) for 15–20 min and incubated with Klotho (Product # PA5‐88303), FoxO1 (ab52857)，Phospho‐FoxO1 (Ser256) (CST #9461), IL‐6 (ab 6672, Abcam), γH2AX (ab 11174)， PARP‐1 (CST #9532), p16INK4a (ab 81278), peroxisome proliferator‐activated receptor‐α (PPARα; sc‐398394) and GAPDH at 4°C overnight. Then, membranes were washed in TBST and incubated with the appropriate secondary antibodies at room temperature for 1 h. Subsequently, membranes were washed in TBST and visualized using the Omni‐ECL basic chemiluminescence detection kit (Shanghai Ya Enzyme Biomedical Technology Co., Ltd.). The housekeeping protein GAPDH was used as loading control.

### 2.10 Enzyme‐linked immunosorbent assay (ELISA)

A total of 59 human serum samples were tested in the ELISA assay. They were categorized into two groups according to the age of individuals: Young (*N* = 23, aged 20–30), and Old (*N* = 36, aged ≥60). The serum concentrations of Klotho (ELISA mskbio [KE10007]) was examined using ELISA kits according to the manufacturer's instructions. After 3 h of incubation at 37°C, the optical density of each well was determined using a microplate reader at 450 nm. For each assay, the serum sample was diluted in a range of 1:1–1:100 into sample diluents. Duplicate assays were performed for each sample. The levels of Klotho (Cusabio, CSB‐E14362m), GDF15 (Cusabio, CSB‐EL009345MO) and CXCL9 in mice serum were quantified using ELISA kits (Cusabio, CSB‐EL006252MO) according to the manufacturer's protocol.

### 2.11 Haematoxylin–eosin and Masson's trichrome staining

The mouse vascular tissues were fixed in 4% paraformaldehyde (Beijing Solarbio Technology Co., Ltd.) at room temperature for 24 h and then embedded in paraffin and cut into 4‐μm‐thick sections. Haematoxylin and eosin and Masson's trichrome staining were used to visualize the vascular architecture and vascular fibrosis, respectively. The slices were examined under an inverted light microscope (Leica). The degree of vascular fibrosis was quantified by ImageJ software. The degree of vascular fibrosis is expressed as a percentage of the fibrotic area of the whole region.

### 2.12 Immunohistochemistry

Four‐micrometre‐thick paraffin sections were deparaffinized in xylene and sequentially rehydrated using a graded series of ethanol. 100°C EDTA (pH = 9) for 20 min. After rinsing in 1× PBS (Leica) for 15 min (three times, 5 min each time), the sections were blocked with 3% hydrogen peroxide for 10 min. Rinse again with 1× PBS for 15 min. The sections were incubated with cleaved PPARα, FoxO1, eNOS, klotho antibody (1:100) overnight at 4°C. Then, the sections were rinsed with PBS for 15 min and incubated with the appropriate secondary antibodies (Leica) for 15 min at RT. After rinsing with 1× PBS for 15 min, immunoreactivity was detected with 3,3′‐diaminobenzidine (DAB) substrate (Leica) for 10 min and the samples were washed with 1× PBS for 15 min. Haematoxylin dye solution was added to the stain at room temperature for 3 min, and the slides were mounted with neutral gum. All the above steps were performed on Leica Bond RX (Leica). The sections were then viewed by using microscopy (magnification 400×; Leica).

### 2.13 Immunofluorescence

Four‐micrometre‐thick paraffin sections were degreased in xylene and repaired sequentially with graded series of ethanol. 100°C EDTA (pH = 9) for 20 min. After rinsing in 1×PBS (Leica) for 15 min (three times for 5 min each), the sections were closed for 10 min at room temperature by adding closure solution and rinsed again with 1× PBS for 15 min. The sections were incubated with FoxO1 and klotho antibodies (1:200) for 60 min at room temperature, then rinsed with PBS for 15 min, and each section was incubated with 150 μL of 1× PBS diluted fluorescent secondary antibody (Nakasugi Jinqiao ZF0311) for 60 min at room temperature. DAPI was added dropwise, incubated for 8 min at room temperature, and then sealed. The sections were observed and scanned in a microscope slide scanner (3DHISTECH Pannoramic 250 FLASH).

### 2.14 Statistical analysis

All data are reported as mean ± SD. Each experiment was repeated at least three times independently. The differences in mean values between the two groups were assessed by the Student's *t*‐test. Statistically significant differences were evaluated at *p* < 0.05.

### 2.15 Potential target identification

The target prediction of the parishin was performed by searching the TCM Systems Pharmacology (TCMSP, https://tcmspw.com/tcmspsearch.php)^15^ BATMAN‐TCM (a Bioinformatics Analysis Tool for Molecular mechanism of Traditional Chinese Medicine，http://bionet.ncpsb.org.cn/batman‐tcm/)^16^ and Swiss Target Prediction (https://www.swisstargetprediction.ch/) databases.^17^ All targets were combined, and repetitive targets were eliminated.

### 2.16 Screening common targets between vascular ageing and parishin

The targets related to vascular ageing were obtained in papers from Human Aging Genomic Resources (HAGR),^18^ Aging Atlas^19^ and CellAge^20^ databases. The common targets between the parishin and vascular ageing were collected for further study.

### 2.17 Enrichment analysis and construction of PPI network

Metascape (https://metascape.org),^21^ an effective and efficient web‐based platform designed to provide a comprehensive gene list annotation and enrichment analysis, was utilized for Gene Ontology (GO)^22^ as well as Kyoto Encyclopedia of Genes and Genomes (KEGG)^23^ pathway. The GO project provides a set of hierarchical controlled vocabulary to identify potential biological mechanisms based on the high‐throughput sequencing genomic data, which split into three categories: BP, molecular function (MF), cellular component (CC). In addition, KEGG pathway database includes a collection of pathway maps representing knowledge on the molecular interaction and reaction networks for metabolism, genetic information processing, cellular processes and human diseases. In this study, the enrichment terms with an FDR < 0.05 were selected, and only the top 30 GO biological functions or KEGG pathways were displayed.

The common targets were imported into the String database (https://string‐db.org/) to obtain the protein–protein interaction (PPI) network when the species was set to homo sapiens. The result was saved as TSV format file which included Node 1, Node 2 and combined score‐related information. The data with a combined score >0.7 were inputted into Cytoscape (Version 3.6) to create a PPI network.

### 2.18 Molecular docking

The interaction between the parishin and corresponding vascular ageing targets were further measured by AutoDock Vina software.^24^ The three‐dimensional structure of the target protein and the chemical structure of parishin were obtained from RCSB PDB database and PubChem database, respectively. The ChemOffice software was used to build the 3D structure of parishin and minimize its energy. The 3D structure of the target protein in PDB format was downloaded, and the operation of dehydrating and hydrogenation of the protein was carried out by using PyMol software. The format of the active component and target protein was converted into PDBQT format by using AutoDock software. Finally, Vina was run for docking.

## 3 RESULTS

### 3.1 Parishin delays HCAEC replicative senescence

**FIGURE 1:**
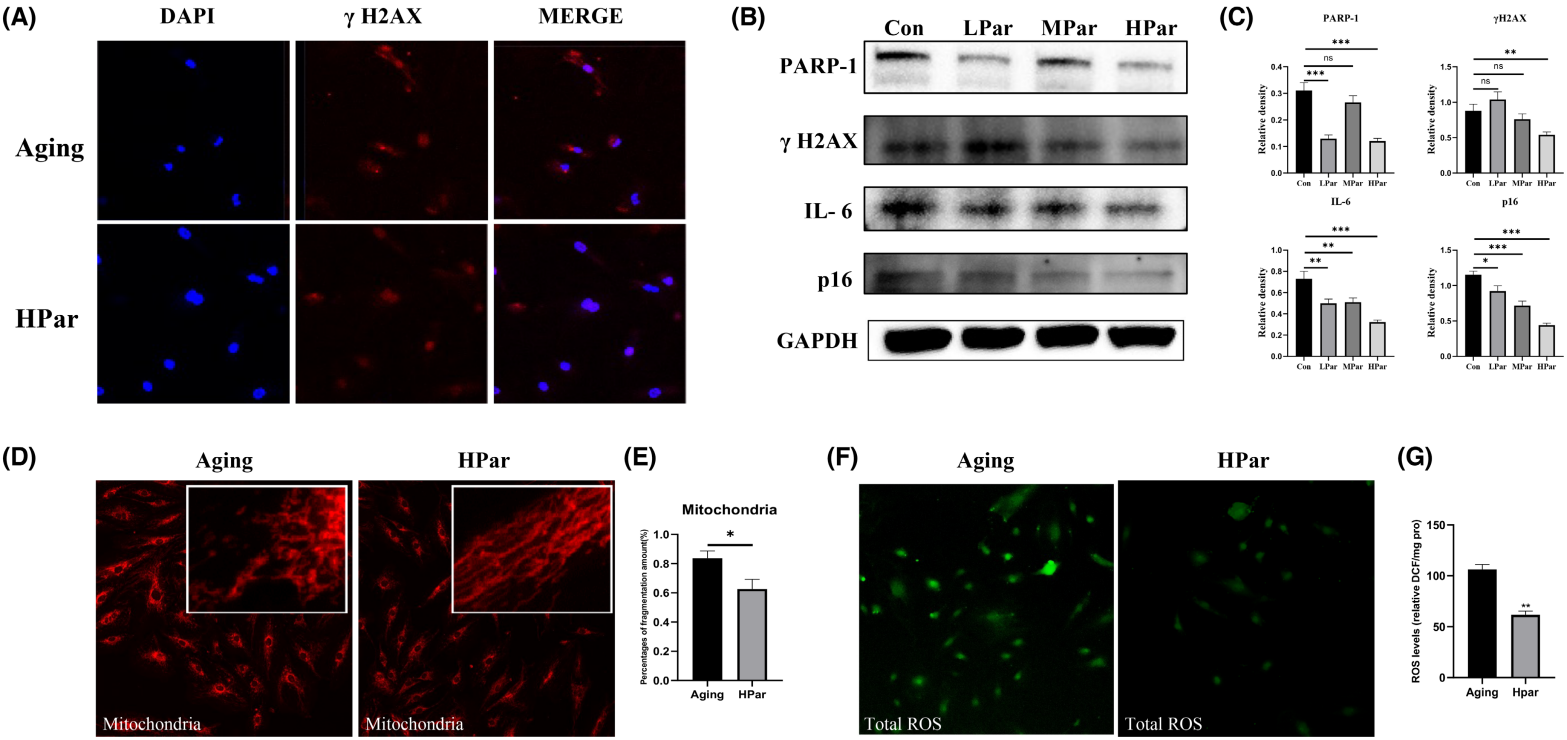
Parishin delays HCAEC replicative senescence. (A) Immunofluorescence staining of γH2AX expression in the high‐dose parishin intervention groups under the indicated conditions. Scale bar, 10 μm. (B) Western blot was used to examine the expression of PARP 1 γH2AX, IL‐6 and p16^Ink4a^ protein expression in replicative senescent cells among different groups. (C) Differences in mitochondrial morphology between the high‐dose parishin intervention group and the elderly control group. (D) Differences in total ROS expression between the high‐dose parishin intervention group and the elderly control group. (E) The corresponding quantitative data of ROS expression. **p < 0.01, versus the control group.

To investigate parishin effect on HCAEC, we successfully constructed a replicative senescence model of HCAEC by natural passaging (Figure S1A,B). We calculated the number of SA‐β‐galactosidase staining‐positive cells from P13 to passage 16 (P16), found HCAEC appeared 4% SA‐β‐galactosidase staining‐positive cells and approached replicative senescence at P16. Therefore, we used the 13th passage (P13) of HCAEC as the starting point for parishin intervention. HCAEC were continuously cultured with low (LPar) and high (HPar) doses of parishin up to the 16th passage (P16), while the control group was given a drug‐free culture medium. DNA damage response/repair factor, senescent biomarker and SASP, such as PARP1, γH2AX, IL‐6, and p16^Ink4a^, were examined at P16.^25^, ^26^, ^27^, ^28^ γH2AX fluorescence signal was reduced in parishin intervention groups compared with the control group (Figure 1A). As determined by immunoblotting, parishin treatment significantly reduced DNA damage response/repair factors, senescent markers and inflammatory factors including PARP1, γH2AX, IL‐6 and p16^Ink4a^ (Figure 1B,C). Previous evidence suggested parishin had antioxidant function in yeast.^7^ To assess the effect of parishin on mitochondria function in senescent HCAEC, we used the mitochondrial fluorescent probe labelling method to observe the structural alterations of mitochondria under confocal microscopy. The results showed that parishin improved morphological mitochondria compared to the control with swollen, elongated, disordered ridge arrangement or fracture (Figure 1D). We quantified percentages of fragmentation amount for mitochondria. The cells containing fragmented mitochondria and those with filamentous mitochondria were counted to calculate the percentage of cells with mitochondrial fragmentation. We found parishin reduced mitochondria fragmentation (Figure 1E).

Moreover, we measured the total ROS by fluorescent probe DCFH‐DA and found the total ROS level was significantly decreased in HPar groups (Figure 1F,G). Collectively, these observations demonstrated parishin treatment alleviated DNA damages, reduced senescent markers and inflammatory factors, simultaneously decreased ROS pressures in senescent HCAEC.

### 3.2 Parishin reduces senescent markers of vascular tissues and serum in naturally aged mice

**FIGURE 2:**
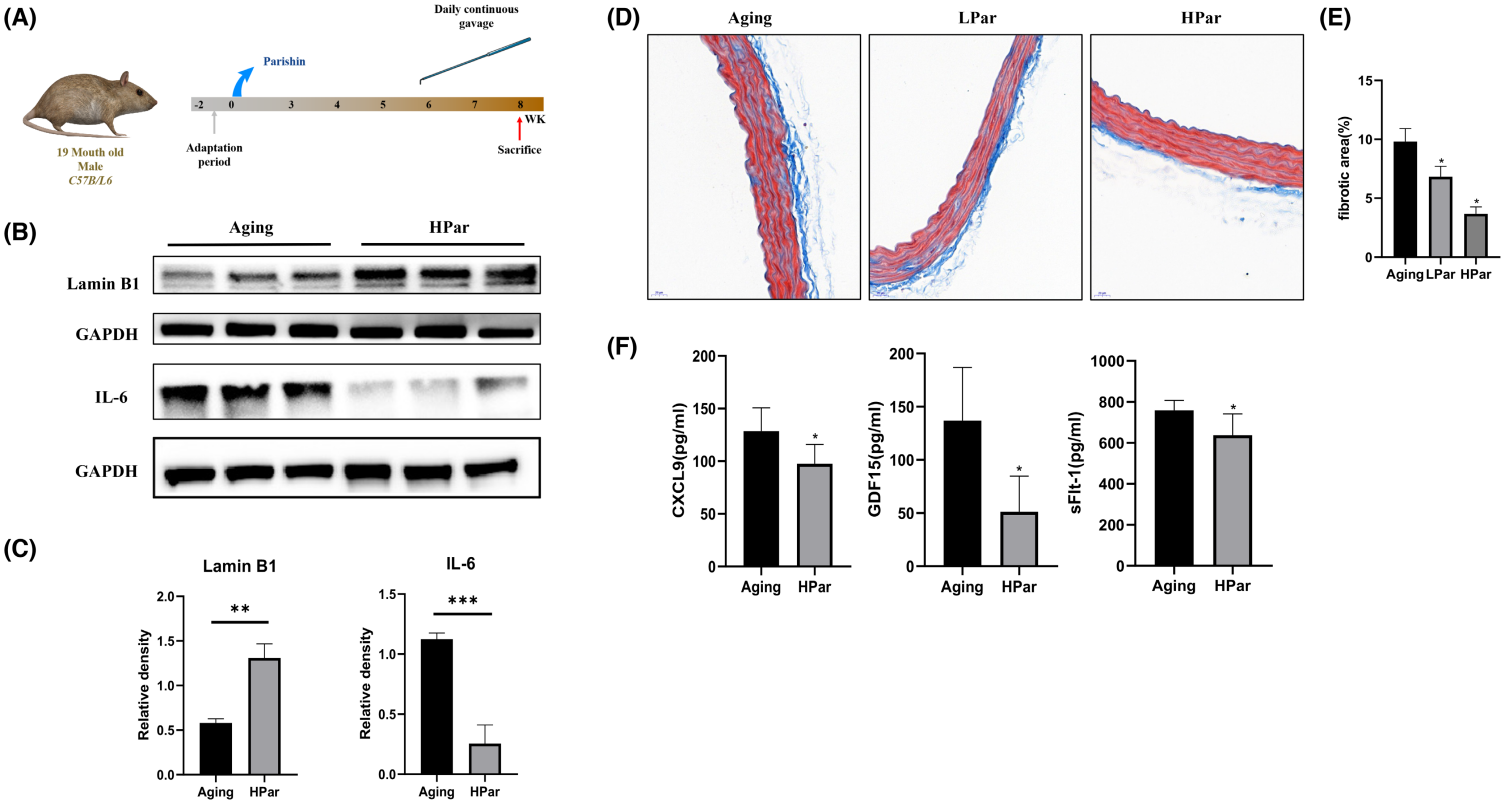
Parishin reduces senescent markers of vascular tissues and serum in naturally aged mice. (A) Temporal schematic diagram of the experimental procedures. (B, C) Western blotting and quantification of lamin B1and IL‐6 in vascular tissue of different groups. (D) The degree of vascular fibrosis was investigated by Masson's trichrome staining (magnification 40×). (E) The corresponding quantitative data of Masson's trichrome staining. (F) Serum GDF15, CXCL9 and sFlt1 levels in different groups were measured using ELISA kits. *p < 0.05 versus Control mice.

To examine the function of parishin suppress vascular ageing in vivo, we chose naturally aged C57B/L6 mice (19 months, 20–30 g). The aged mice were randomly divided into three groups (*N* = 10), ageing control (AC), low dose (LPar, 10 mg/kg/day) and high dose (HPar, 20 mg/kg/day). Parishin was dissolved in 0.9% saline and administered as a suspension by the gavage method. Treatment was continued for 8 weeks, once daily (Figure 2A). Lamin B1 loss was found in senescence and led to changes of nuclear size and shape.^29^ Parishin upregulated lamin B1 in vascular tissue (Figure 2B,C). There was a significant reduction in IL‐6, one of the SASP factors, by parishin treatment compared to the control group (Figure 2B,C). Tissue histopathology staining by Masson was performed on vascular tissue to determine vascular texture. The results showed that the wall of aorta in the elderly group was thick, especially the middle layer of vascular smooth muscle cells. The elastic fibre plate was also destroyed and became disorganized in the elderly group. Meanwhile, the collagen fibres were diffusely increased and unevenly distributed. Collagen fibres in the endothelium of thoracoabdominal aorta was reduced depending on different doses of parishin, as shown in Figure 2D,E. It was suggested that parishin treatment improved vascular texture in naturally aged mice. To further assess the effect of parishin on vascular ageing, we examined serological indicators related to ageing and vascular function. Growth differentiation factor 15 (GDF15) and CXCL9 are associated with inflammation and thought to be a stress‐inducing factor.^30^, ^31^, ^32^ CXCL9 is a master regulator of vascular function and cellular senescence. Senescent endothelial cells in human and mice show vascular dysfunction, which is reversed by silencing CXCL9.^33^ Age‐related increases in sFltl expression in the aorta, central hepatic vein, hepatic sinusoidal cells and muscle capillaries were identified. Serum GDF15, CXCL9 and sFlt1 levels in different groups were measured using ELISA kits. As shown in Figure 2F, expression of GDF15, CXCL9 and sFlt1 was significantly decreased in the parishin treatment groups.

### 3.3 Parishin improves endothelial cell function in naturally aged mice

**FIGURE 3:**
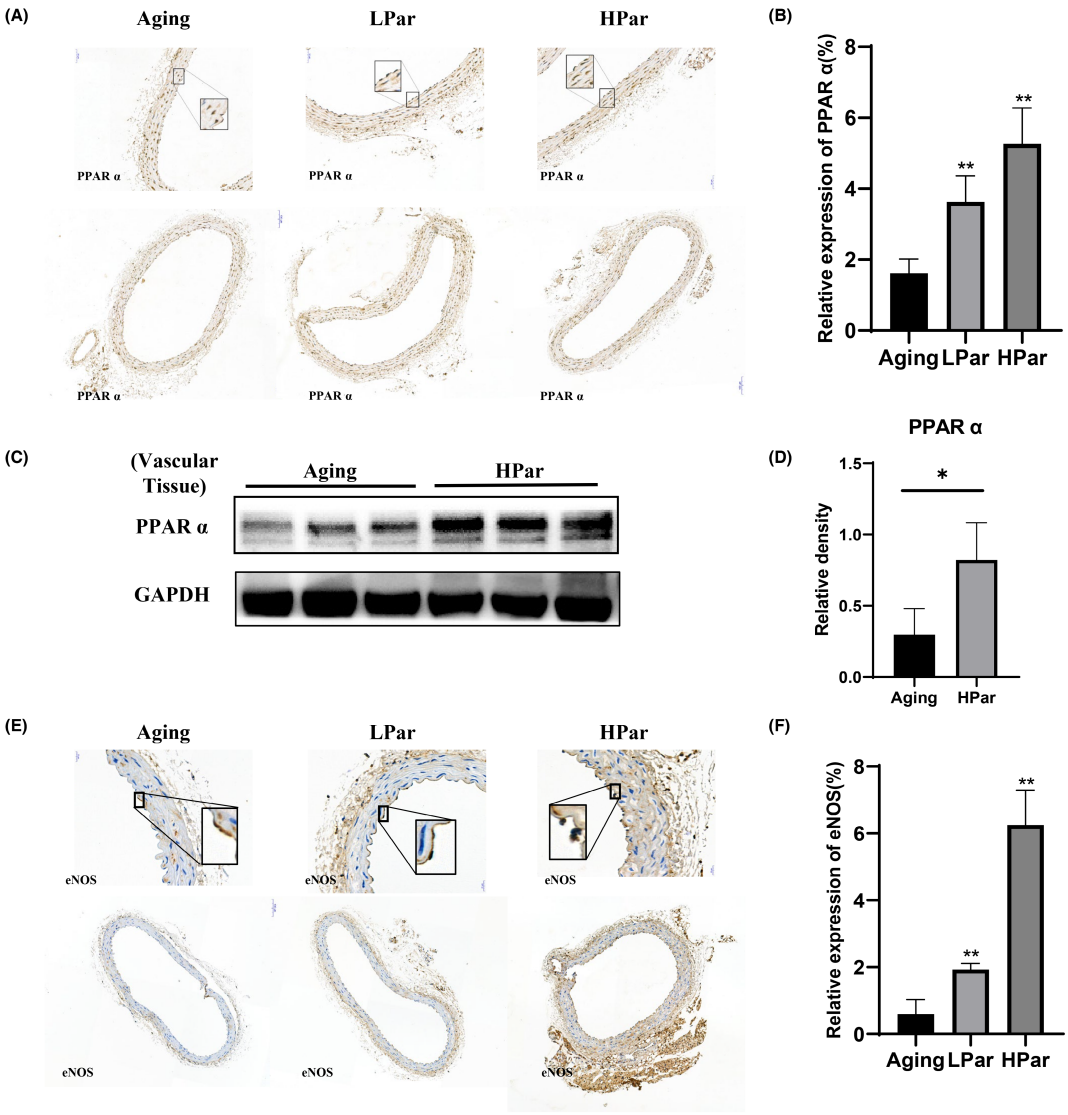
Parishin improves endothelial cell function in naturally aged mice. (A) Immunohistochemistry results revealed the expression of PPARα in different groups (magnification 20×). (B) The corresponding quantitative data analysis of PPARα expression was done with ImageJ software. (C, D) Western blot was used to examine the expression of PPARα protein in each vascular tissue. (E) Immunohistochemistry results revealed the expression of eNOS in different groups (magnification 40×). (F) The corresponding quantitative data analysis of eNOS expression was done with ImageJ software. ** *p* < 0.01, versus the control group. * *p* < 0.05, versus the control group.

PPARα is reduced in aged mice which impaired cardiovascular system.^34^, ^35^ PPARα activators stimulate the production of nitric oxide by increasing endothelial nitric oxide synthase (eNOS).^36^ It is known that nitric oxide production by endothelial cells is particularly important for the maintenance of normal vascular function. In senescent human endothelial cells, eNOS activity and nitric oxide production are impaired.^37^ Hutchinson–Gilford progeria syndrome endothelial cells show reduced eNOS expression.^38^ To evaluate parishin effects on endothelial cell function, both immunohistochemical and WB assays were performed on vascular tissues. We found that parishin treatment stimulated the expression of PPARα (Figure 3A–D). Immunohistochemical experiments revealed that eNOS expression was upregulated in parishin treatment groups compared with control groups, especially the HPar group (Figure 3E,F). Taken together, this new observation suggested parishin has the potential to protect vascular vessels by increasing PPARα and eNOS expression in naturally aged mice.

### 3.4 Parishin increases α‐Klotho of vascular tissue in naturally aged mice

To determine what is parishin target, we conducted targets prediction based on TCMSP, BATMAN‐TCM and Swiss Target Prediction databases. A total of 595 targets were gathered after removing duplicates. Meanwhile, 506 vascular ageing targets were retrieved and integrated through data mining using the HAGR, Aging Atlas and CellAge databases. The ‘Common targets’, intersected by component targets and vascular ageing targets, were regarded as the target genes responsible for the vascular ageing effect of parishin. Then, 57 common active ingredient disease targets at the intersection were further screened (Figure S2A).

The enrichment analysis results showed that most common genes were particularly enriched in BP, including transcription from RNA polymerase II promoter, NF‐kappa B transcription factor activity and transcription, DNA template. Major enrichment in CC included nucleoplasm, cytosol and extracellular space. Primary enrichment in MF consisted of transcription factor binding, enzyme binding and cytokine activity (Figure S2B). KEGG pathway analysis revealed that the targets were mainly enriched in the PI3K‐Akt signalling pathway, Pathways in cancer, MAPK signalling pathway, Ras signalling pathway and endocrine and other factor‐regulated calcium reabsorption (Figure S2C). To further identify key targets in a network background, a PPI network was constructed based on common targets retrieved from the String database (https://string‐db.org/cgi/input.pl). Cytoscape software 3.6 (https://cytoscape.org/) was utilized to analyse topological data to determine the accuracy of the critical targets and the reliability of the virtual prediction of the molecular docking method. As seen in Figure S2D, the PPI network comprised 52 nodes and 325 edges.

Molecular docking was used to further verify the accuracy of the key targets. We used KL (PDB ID: 5W21) as the test protein target to verify the accuracy. The docking results of the target protein Klotho protein with parishin are shown in Figure S2E. Amino acid residues ILE‐836, TRP‐838, GLY‐878, GLN‐396 and ASN‐304 in the crystal structure of Klotho formed hydrogen bonds with parishin. The docking scores for the parishin with the Klotho crystal structure were −8.1 kcal/mol. The docking score represents the binding affinity, and when the score is lower, the binding affinity is stronger. An affinity <−7 kcal/mol indicates strong binding activity. Our analysis indicated that the parishin had a strong binding affinity for Klotho.

**FIGURE 4:**
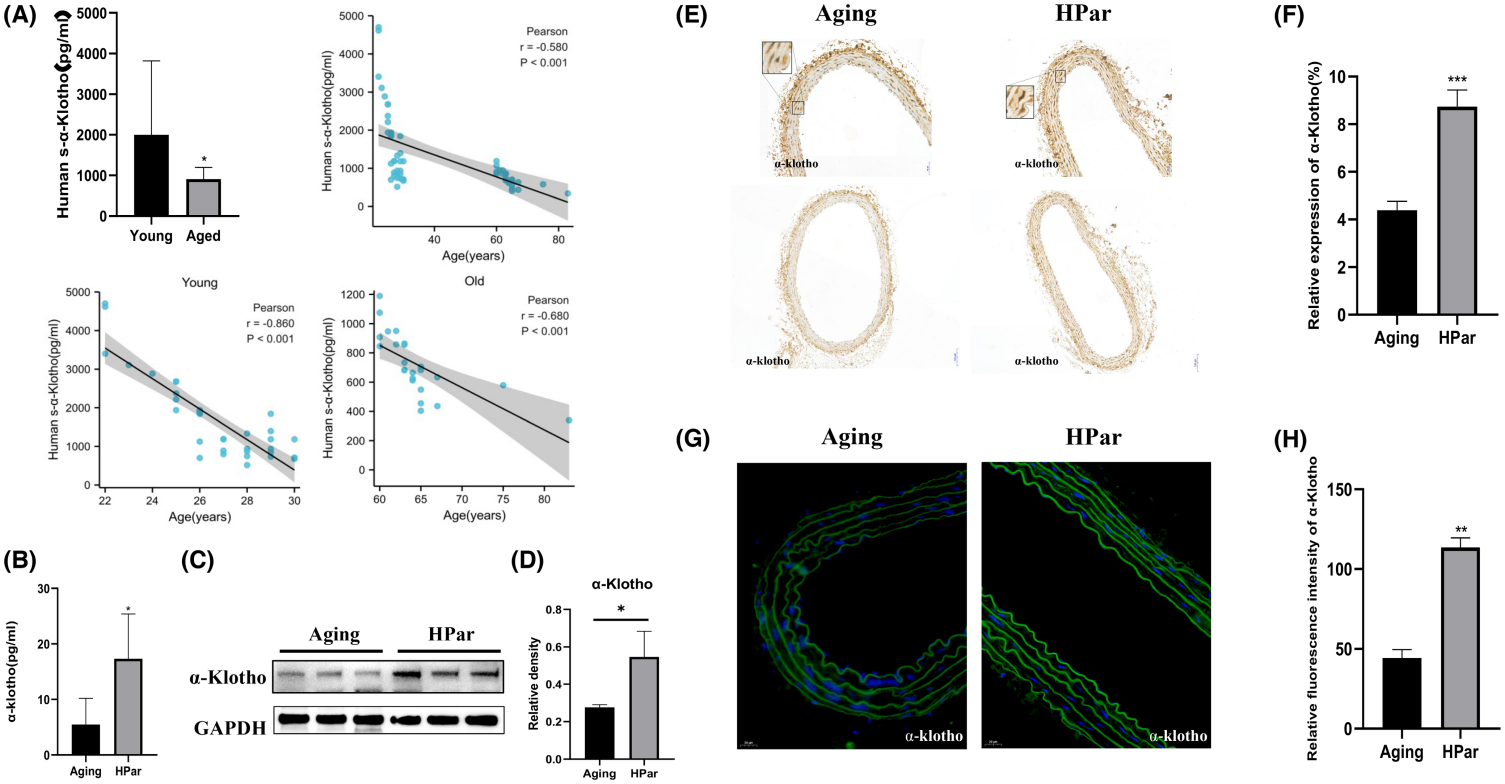
Parishin increases α‐Klotho of vascular tissue in normal ageing mice. (A) The human serum α‐Klotho levels among the Young and Old groups were measured using ELISA. Pearson's correlation analysis of α‐Klotho levels based on age. (B) Serum α‐Klotho levels in different ageing mice groups were measured using ELISA kits. (C) Western blot was used to examine the expression of α‐Klotho protein in each vascular tissue. (D) Immunohistochemistry results revealed the expression of α‐Klotho in different groups (magnification 20×). (E) The corresponding quantitative data analysis of α‐Klotho expression was done with ImageJ software. (F) Immunofluorescence staining of α‐Klotho expression in the high‐dose parishin intervention groups under the indicated conditions. Scale bar, 20 μm. (G) The corresponding quantitative data analysis of α‐Klotho expression was done with ImageJ software. ***p < 0.001, versus the control group. **p < 0.01, versus the control group. *p < 0.05, versus the control group.

α‐Klotho is a geroprotective protein that prevents functional ageing of vascular endothelial cells, regulates vascular development, protects myocardium and resists atherosclerosis through various mechanisms.^11^, ^39^, ^40^ To understand the relationship between α‐Klotho and age, we first measured human serum α‐Klotho levels using ELISA. We observed different human serum α‐Klotho levels among Young and Old groups (Figure 4A). Serum α‐Klotho level in elderly adults was significantly decreased comparing to young adults. To further verify the results mentioned above, we performed Pearson's correlation analysis of α‐Klotho levels based on age. α‐Klotho expression was significantly inversely and linearly associated with age (*r* = −0.580) (Figure 4A). Pearson's regression analysis revealed that the α‐Klotho serum level was significantly negatively correlated with age (*r* = −0.860 and −0.680, respectively) (Figure 4A). In conclusion, we found a negative correlation between serum α‐Klotho concentration and ageing in human beings.

Next, we determined whether parishin increases α‐Klotho in normal ageing mice. Indeed, parishin upregulated serum and vascular tissue α‐Klotho in HPar groups using ELISA and immunoblotting, respectively (Figure 4B–D). Immunohistochemical analysis of vascular tissue demonstrated that parishin treatment markedly increased the expression of α‐Klotho, and the difference was statistically significant (Figure 4E,F). Immunofluorescence results also showed that the fluorescence signal of α‐Klotho was enhanced in HPar groups compared with the control group, and the difference was statistically significant (Figure 4G,H). Our results suggested that parishin treatment in vivo is efficient in delaying ageing‐related vascular dysfunction by stimulating α‐Klotho production.

### 3.5 Parishin upregulates FoxO1 in naturally aged mice

**FIGURE 5:**
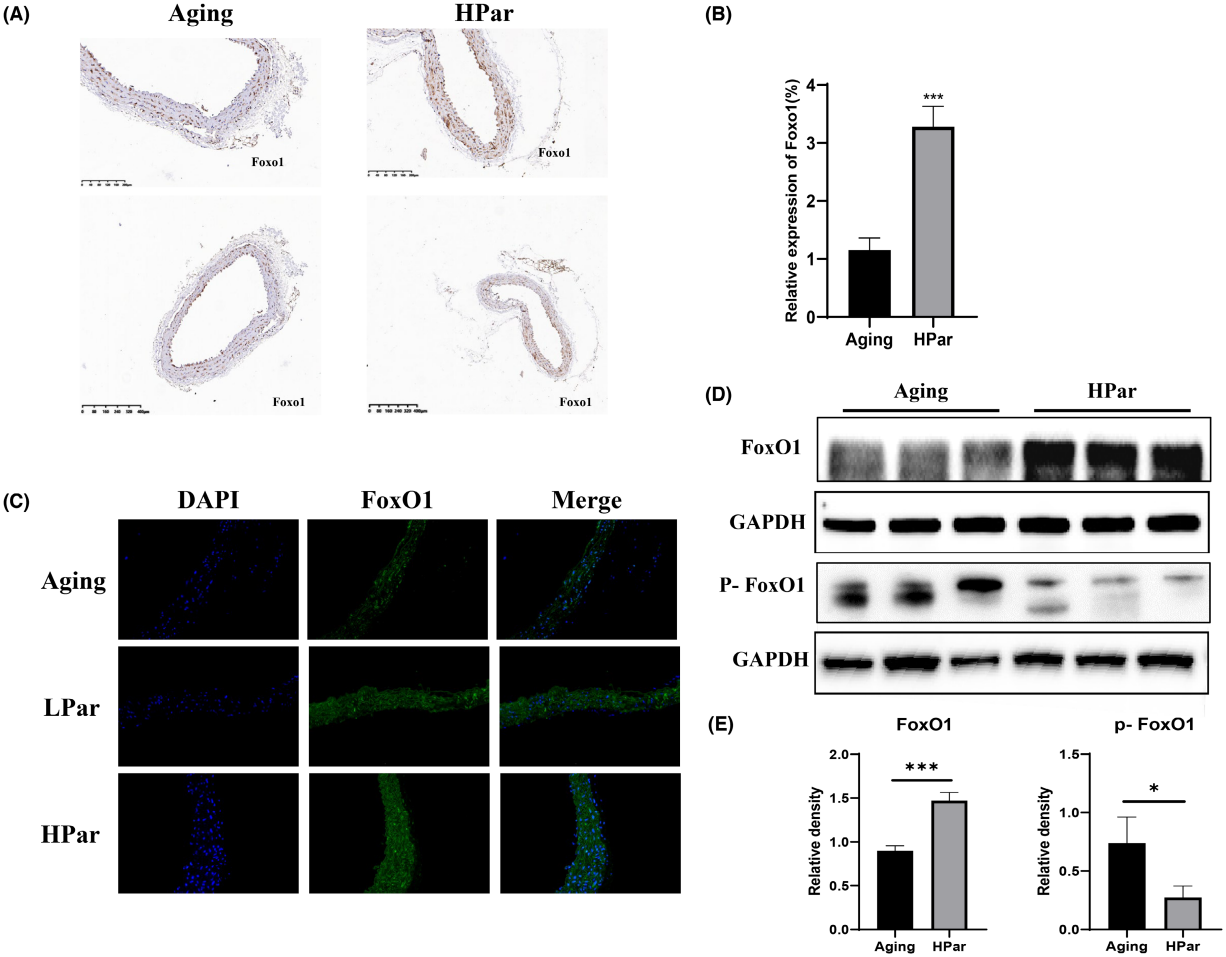
Parishin upregulates FoxO1 in naturally aged mice. (A) Immunohistochemistry results revealed the expression of FoxO1 in different groups (magnification 20×). (B) The corresponding quantitative data analysis of FoxO1 expression was done with ImageJ software. (C) Immunofluorescence staining of FoxO1 expression in different groups, Scale bar, 20 μm. (D, E) Western blot was used to examine the expression of FoxO1 and P‐FoxO1 protein in each vascular tissue. ****p < 0.001, versus the control group. *p < 0.05, versus the control group.

Previous studies have shown that Klotho promoted translocation of FoxO1 from the cytoplasm to the nucleus by reducing FoxO1 phosphorylation and increased FoxO1 expression.^12^, ^14^ We conducted immunohistochemistry, immunofluorescence experiments and immunoblotting to detect FoxO1expression in HPar treatment groups. As shown in Figure 5, the expression of FoxO1 was increased in HPar treatment groups compared with the control group. Phosphorylated FoxO1 was decreased in HPar treatment groups compared to untreated control to promote FoxO1 protein level, as shown in Figure 5D,E. Consistent with the results of vascular tissue, parishin treatment improved senescent HCAEC cells. The expression of Klotho and FoxO1 for senescent HCAEC cells was increased in the parishin intervention groups (Figure S3). Phosphorylated FoxO1 also was decreased compared with untreated controls, as shown in Figure S3B. Our experiments demonstrated that parishin treatment led to an upregulation of FoxO1, supporting the Klotho/FoxO1 signalling pathway.

## 4 DISCUSSION

Ageing‐related processes occur at different levels of the biological hierarchy, not only at the cellular level, but also at organ support systems.^41^, ^42^ Vascular ageing is the process by which the structure and function of blood vessels undergo degenerative changes with increasing age. In addition, vascular ageing is an important pathological and physiological basis for the ageing of all human organ systems and is a common pathogenesis of many chronic diseases in the elderly such as hypertension, coronary heart disease and stroke.^43^ Therefore, innovative anti‐ageing interventions are important for prolonging healthy lifespan and delaying vascular lesions caused by ageing.^44^, ^45^, ^46^ In this study, the expression of senescent biomarkers such as IL‐6, γH2AX and p16^Ink4a^was found to be significantly reduced in a dose‐dependent manner in the parishin intervention HCAEC group (Figure 1). In vivo, we tried to detect γH2AX and p16, but could not find the signals in vascular tissues by western blots. In consideration of very little vascular tissues per mouse and avoiding them running out, we determined the level of IL‐6 and lamin B1, the degree of vascular fibrosis by Masson's trichrome staining and the serum level of GDF15, CXCL9 and sFlt1(Figure 2). We found parishin reduced IL‐6, upregulated lamin B1 and improved vascular texture in the parishin treatment groups. Interestingly, serum GDF15, CXCL9 and sFlt1 levels were significantly decreased in parishin intervention groups, further supporting that parishin has an anti‐ageing function in the vascular system. In addition, the expression of eNOS, which is closely related to endothelial function and maintains the normal vascular function, was statistically significant (Figure 3E,F). All of these suggest that parishin has a role in protecting vascular tissue from senescence and maintain vascular function.

Vascular ageing is influenced by various factors including genetic, endocrine, metabolic and external environment.^47^ The mechanisms of vascular senescence involve genomic instability, telomere shortening, epigenetic alterations, loss of proteostasis, dysregulation of nutrient perception, mitochondrial dysfunction, cellular senescence, stem cell failure and altered intercellular communication.^45^ Previous studies have found that parishin delays the lifespan of yeast by regulating oxidative stress. Our data showed that the mitochondrial morphological structure was improved in the parishin intervention group with a decrease in the total expression of ROS (Figure 1D–G) and an increase in the expression of PPARα, a mitochondria‐related functional protein, in a dose‐dependent manner (Figure 3A–D). In conclusion, parishin plays an important role in improving mitochondrial function and slowing down ageing by anti‐oxidative stress.

**FIGURE 6:**
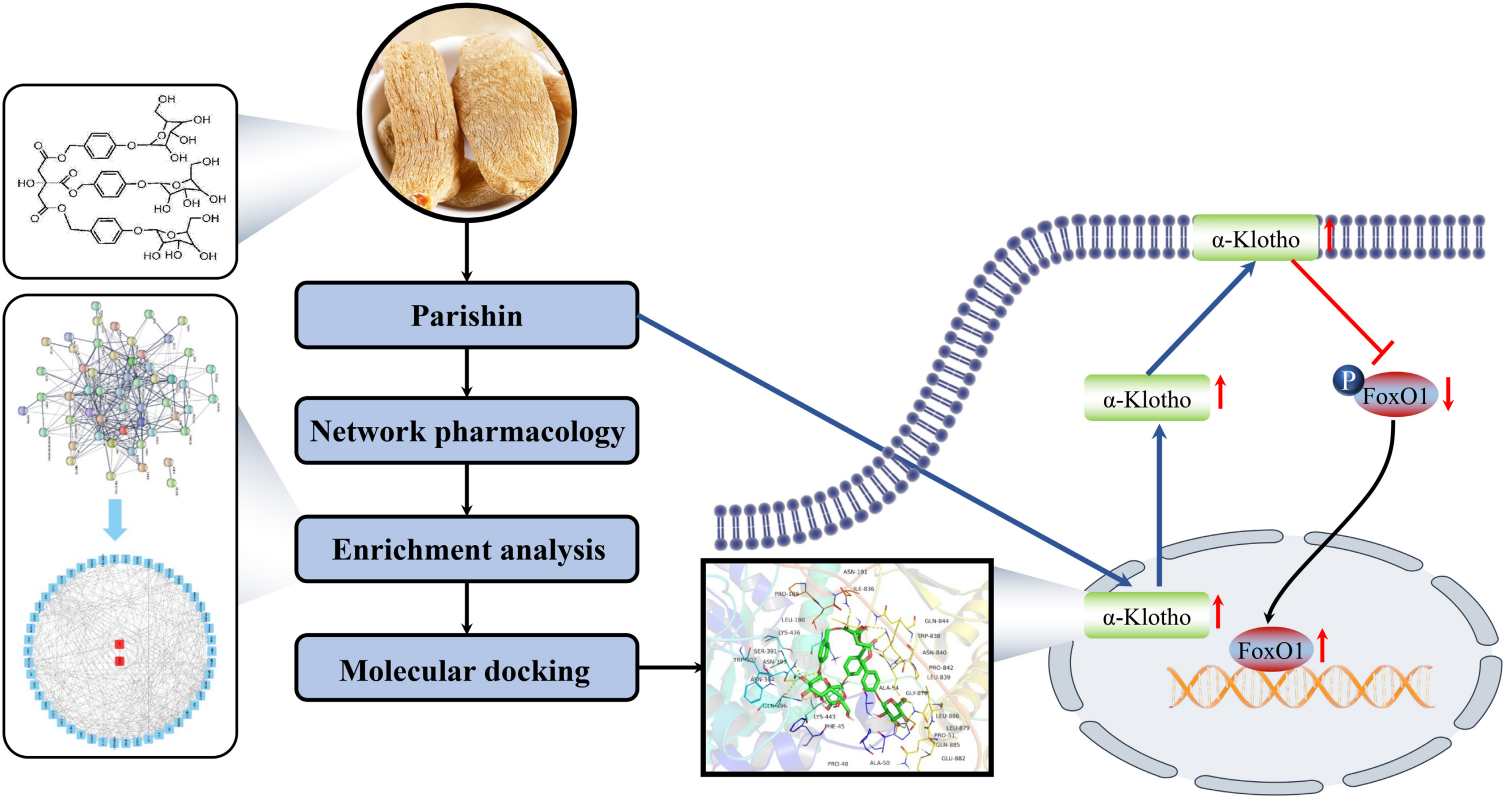
Summary of the molecular mechanism of the anti‐ageing effect of parishin. Parishin treatment reduced the production of ROS, further improved mitochondrial function, then activated the Klotho/FoxO1 signalling pathway and finally exerted the anti‐ageing effect.

Lin et al. found that parishin increased Sir2 gene expression and inhibited the UTH1/TOR signalling pathway in yeast. We found SIRT1 and SIRT6 were significantly upregulated in a dose‐dependent manner in the parishin intervention group by a dose‐dependent manner compared with the control group (Figure S4). In order to further explore parishin targets for delaying vascular ageing, we used a network pharmacology approach to construct a ‘monomer–target–pathway’ network of Chinese herbal extracts to screen the mechanistic targets of parishin. The molecular docking results showed that parishin bound well with Klotho. Currently, Soluble Klotho is considered as an anti‐ageing protein. Lack of Klotho expression in mice produces premature ageing and age‐related diseases, including vascular diseases. Overexpressing Klotho lived longer and did not develop age‐related diseases in animals. In this study, a comparative analysis of human serum soluble Klotho collected at different ages showed an age correlation for Klotho (Figure 4A). The results provided evidence for Klotho as a clinical marker of ageing. Previous studies have found that secretory Klotho induces nitric oxide production in endothelial cells, which allows these cells to withstand oxidative stress. In addition, it has been shown that Klotho protein interacts with the VEGFR‐2/transient receptor potential typical‐1 (TRPC‐1) complex on the surface of endothelial cells.^40^ This molecular complex is incorporated into the cells and is involved in stabilizing Ca^2+^ entry to maintain the integrity of endothelial tissue. Soluble Klotho thus protects endothelial cells from SASP. Similarly, the present study found that serum soluble ageing factors were decreased and vascular tissue eNOS expression increased in ageing mice after parishin intervention compared with controls (Figures 2F and 3E). Subcellular localization and transcriptional activity of FoxO1 are regulated by post‐translational modifications (e.g. phosphorylation), which inhibit FoxO1 by inducing cytoplasmic localization and proteasomal degradation activity of FoxO1.^48^ FoxO1 in the nucleus directly binds to the promoter of the antioxidant stress enzyme and exerts antioxidant effects.^49^ In this study, both cellular and animal experiments showed that parishin intervention group was higher protein level of Klotho and FoxO1 than the control group (Figures 4 and 5). The results showed that parishin exerted an anti‐oxidative stress effect. In conclusion, we tentatively found that parishin is a valuable monomer that protects vascular endothelial cells from senescence via the Klotho/FoxO1 signalling pathway (Figure 6). Given the present results, parishin may prove to be useful against endothelial cell senescence, premature vascular ageing and related complications.

In this study, to investigate the parishin function at early intervention on HCAEC, we constructed a replicative senescence model of HCAEC by natural passaging (Figure S1A,B). SA‐β‐galactosidase staining experiment showed that HCAEC approached replicative senescence at Passage 16. Therefore, we used the 13th passage of HCAEC as the starting point for parishin early intervention and collected the results at Passage 16. We did not examine the levels of p16, IL6 and others of senescent biomarkers in early passage cells (Figure 1) and adult mouse control, and will add early passage cells and adult mouse control in follow‐up studies.

Our data showed that parishin has the potential to intervent the vascular senescence, which will help to further elucidate the mechanism of parishin action in vascular diseases and provide a theoretical basis for the rational clinical use of parishin. In order to clarify the role of parishin in delaying vascular ageing by regulating the Klotho/FoxO1 signalling pathway, our group will conduct further studies on this pathway, as well as future clinical studies to evaluate the efficacy, safety and tolerability of parishin.

## AUTHOR CONTRIBUTIONS

**Yunmei Yang:** Conceptualization (lead); funding acquisition (lead); project administration (supporting); writing – review and editing (lead). **Xinxiu Zhao:** Formal analysis (lead); investigation (lead); methodology (lead); validation (lead); visualization (lead); writing – original draft (lead). **Shixian Zhou:** Data curation (supporting); methodology (supporting); software (supporting); validation (supporting); visualization (supporting). **Yang Liu:** Data curation (supporting); methodology (supporting); software (supporting); validation (supporting); visualization (supporting). **Caixia Gong:** Methodology (supporting); software (supporting); visualization (supporting). **Lan Xiang:** Methodology (supporting); resources (supporting). **Shumin Li:** Methodology (supporting); validation (supporting); visualization (supporting). **Peixia Wang:** Methodology (supporting); software (supporting); visualization (supporting). **Yuejun Wang:** Software (supporting); visualization (supporting). **Dr Linlin Sun:** Project administration (supporting); supervision (supporting); writing – review and editing (supporting). **Qin Zhang:** Funding acquisition (supporting); resources (supporting); supervision (supporting); writing – review and editing (supporting).

## FUNDING INFORMATION

This work was supported by the Project of Traditional Chinese Medicine Science and Technology Plan in Zhejiang Province (nos 2020ZQ032 and 2021ZQ052); The Traditional Chinese Medicine (Integrated Chinese and Western Medicine) Key Discipline Construction Project of Zhejiang Province (no. 2017‐XK‐A31).

## CONFLICT OF INTEREST STATEMENT

The authors declare that they have no conflict of interest.

## Supporting information


Figure S1

Figure S2

Figure S3

Figure S4

## Data Availability

Data openly available in a public repository that issues datasets with DOIs.
