# Supplementary figures and images for: Parishin alleviates vascular ageing in mice by upregulation of Klotho
Source: J Cell Mol Med. 2023 Apr 9;27(10):1398–409. doi: 10.1111/jcmm.17740 (PMC10183705; doi:10.1111/jcmm.17740)

# Supplement Figures

Fig. S1

A

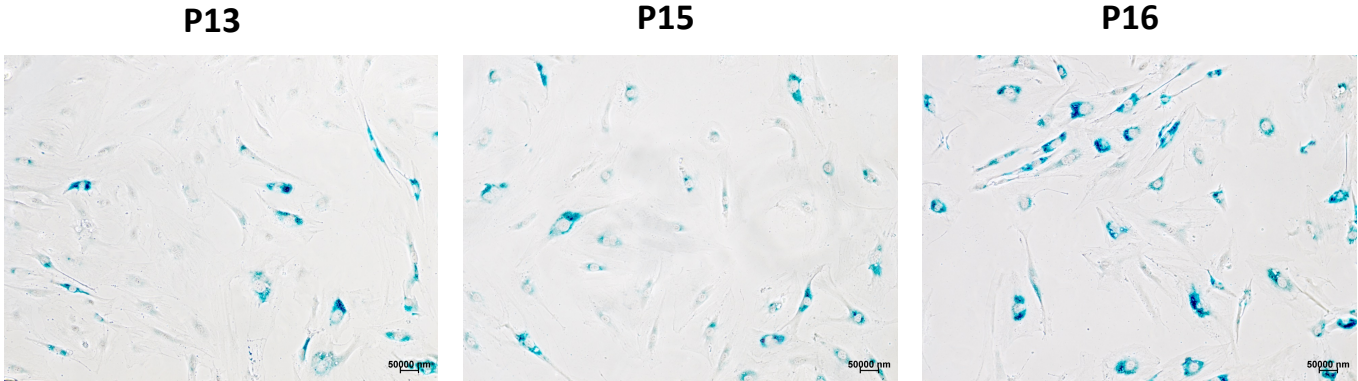

B

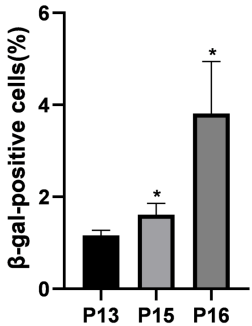

Supplement: Supplementary file 1 — Figure S1 [file JCMM-27-1398-s002.pdf]

Fig. S2

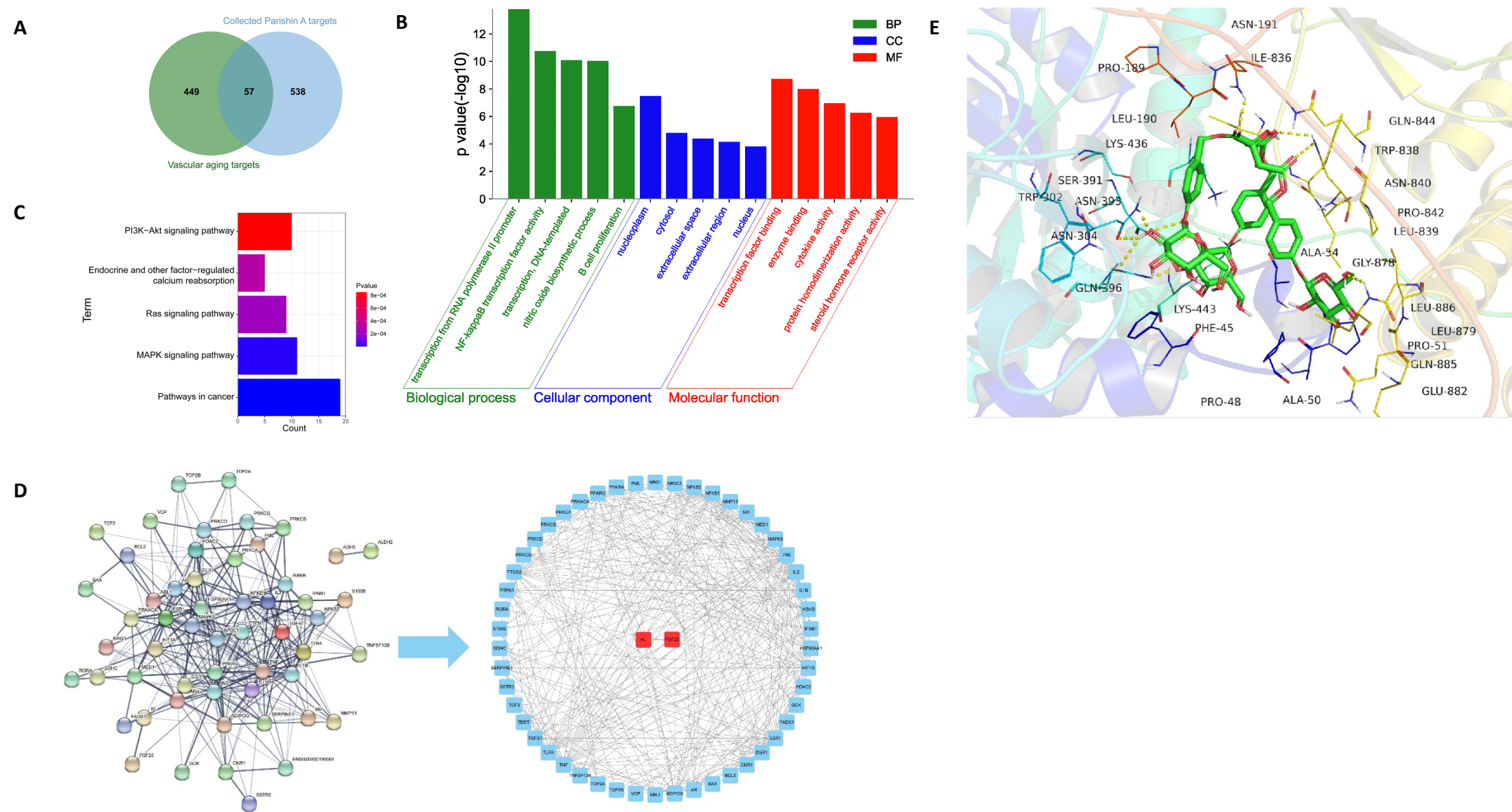

Supplement: Supplementary file 2 — Figure S2 [file JCMM-27-1398-s004.pdf]

Fig. S3

A

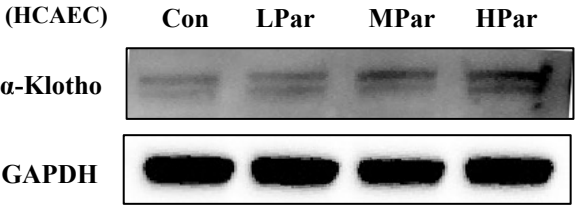

B

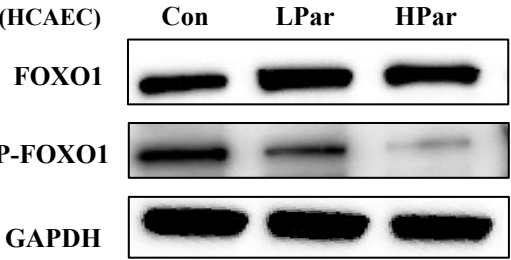

Supplement: Supplementary file 3 — Figure S3 [file JCMM-27-1398-s001.pdf]

Fig. S4

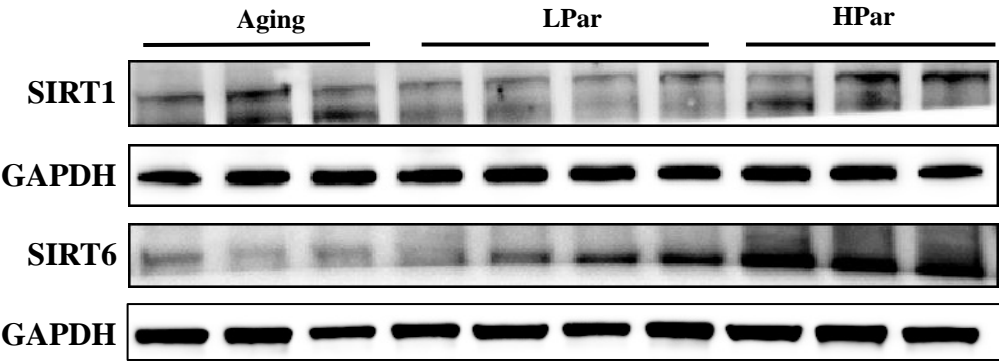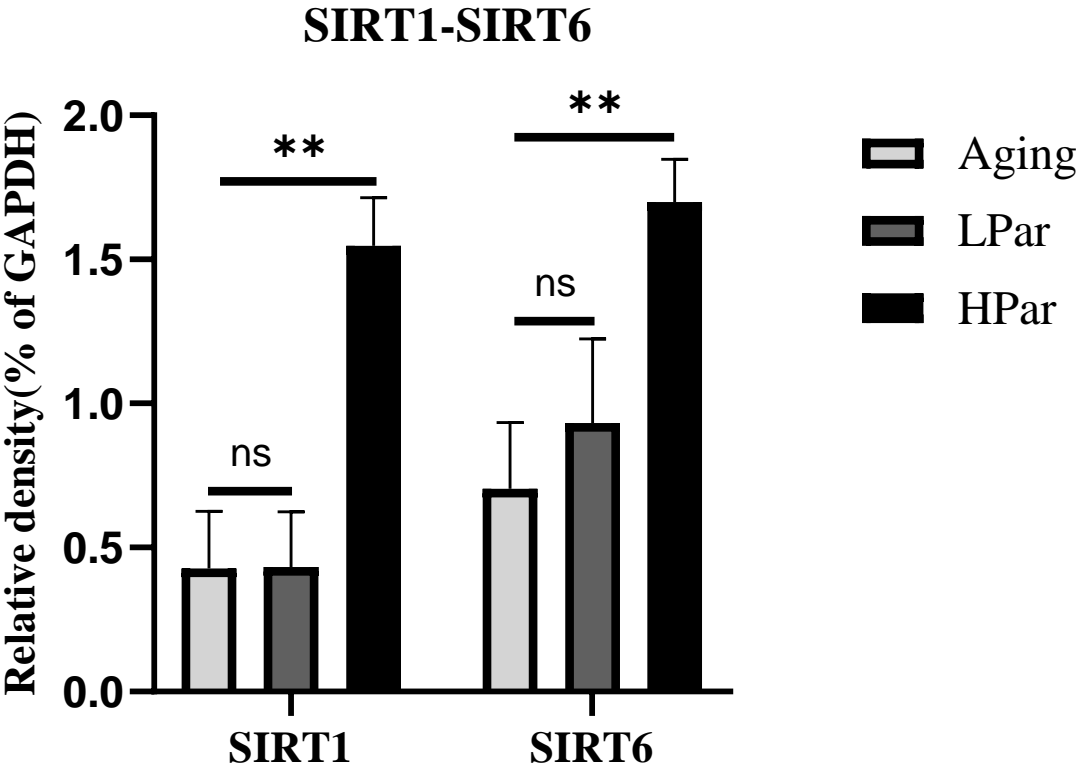

Supplement: Supplementary file 4 — Figure S4 [file JCMM-27-1398-s003.pdf]
